# Assemblies of Coaxial Pick-Up Coils as Generic Inductive Sensors of Magnetic Flux: Mathematical Modeling of Zero, First and Second Derivative Configurations

**DOI:** 10.3390/s24123790

**Published:** 2024-06-11

**Authors:** Petros Moraitis, Dimosthenis Stamopoulos

**Affiliations:** Department of Physics, School of Science, National and Kapodistrian University of Athens, 15784 Athens, Greece; morapet@phys.uoa.gr

**Keywords:** magnetic field sensors, AC magnetic susceptibility, sensitivity of coils, inductive sensors, magnetometers, pick up coils, susceptometers

## Abstract

Coils are one of the basic elements employed in devices. They are versatile, in terms of both design and manufacturing, according to the desired inductive specifications. An important characteristic of coils is their bidirectional action; they can both produce and sense magnetic fields. Referring to sensing, coils have the unique property to inductively translate the temporal variation of magnetic flux into an AC voltage signal. Due to this property, they are massively used in many areas of science and engineering; among other disciplines, coils are employed in physics/materials science, geophysics, industry, aerospace and healthcare. Here, we present detailed and exact mathematical modeling of the sensing ability of the three most basic scalar assemblies of coaxial pick-up coils (PUCs): in the so-called zero derivative configuration (ZDC), having a single PUC; the first derivative configuration (FDC), having two PUCs; and second derivative configuration (SDC), having four PUCs. These three basic assemblies are mathematically modeled for a reference case of physics; we tackle the AC voltage signal, V_AC_ (t), induced at the output of the PUCs by the temporal variation of the magnetic flux, Φ(t), originating from the time-varying moment, **m**(t), of an ideal magnetic dipole. Detailed and exact mathematical modeling, with only minor assumptions/approximations, enabled us to obtain the so-called sensing function, F_SF_, for all three cases: ZDC, FDC and SDC. By definition, the sensing function, $F_{SF}$, quantifies the ability of an assembly of PUCs to translate the time-varying moment, **m**(t), into an AC signal, V_AC_ (t). Importantly, the $F_{SF}$ is obtained in a closed-form expression for all three cases, ZDC, FDC and SDC, that depends on the realistic, macroscopic characteristics of each PUC (i.e., number of turns, length, inner and outer radius) and of the entire assembly in general (i.e., relative position of PUCs). The mathematical methodology presented here is complete and flexible so that it can be easily utilized in many disciplines of science and engineering.

## 1. Introduction

Among all building elements of electrical engineering in general and of nonintegrated and integrated electronics in particular, coils are probably the simplest, cheapest and most effective component, especially when we refer to sensing applications. Many of the advantages of coils lie in the fact that, nowadays, they can be produced in high throughputs by using versatile, highly productive, well-established methods even for the more complicated structures (cylindrical, planar, spiral, conical, etc.) of miscellaneous dimensions (from the range of m down to nm), depending on the specifications of each application. For instance, coils come in a three-dimensional, macroscopic form wound on frames that are either hollow (air core) or enclosing a material of specified magnetic properties used to focus the magnetic flux (magnetic core). Also, coils can be printed equally well on solid planar substrates or the surface of flexible membranes.

A useful characteristic of coils is their bidirectional operation: they can be used both to produce and sense magnetic fields. This property makes coils the sole choice in some important applications (e.g., nuclear magnetic resonance in physics/materials science). Especially when we refer to sensors, coils have the physical advantage of inductively translating an AC variation of magnetic flux into an AC voltage signal [1]. Coils used to sense magnetic fields are most commonly termed “pick-up coils” (PUCs). Assemblies of PUCs are employed in both scalar and vector configurations in, among other scientific and engineering disciplines, physics/materials science (e.g., to record the AC magnetic susceptibility of materials) [2,3,4,5,6], electrical/electronic engineering (e.g., to control the trajectory and focus electron beams, to harvest stray energy from the environment, etc.) [7,8,9,10], geophysics (e.g., to survey anomalies in Earth’s magnetic field, to record the AC magnetic susceptibility of natural materials, etc.) [11,12,13], aerospace (e.g., to survey the magnetic field and sense the attitude, to investigate magnetospheric plasma physics, etc.) [14,15], construction (e.g., to survey the rigidity of reinforcement; see [16] and references therein) and healthcare (e.g., in magnetic resonance imaging to get noninvasive visual access to almost every tissue of the human body and other biomedical applications) [17,18,19,20].

**Figure 1:**
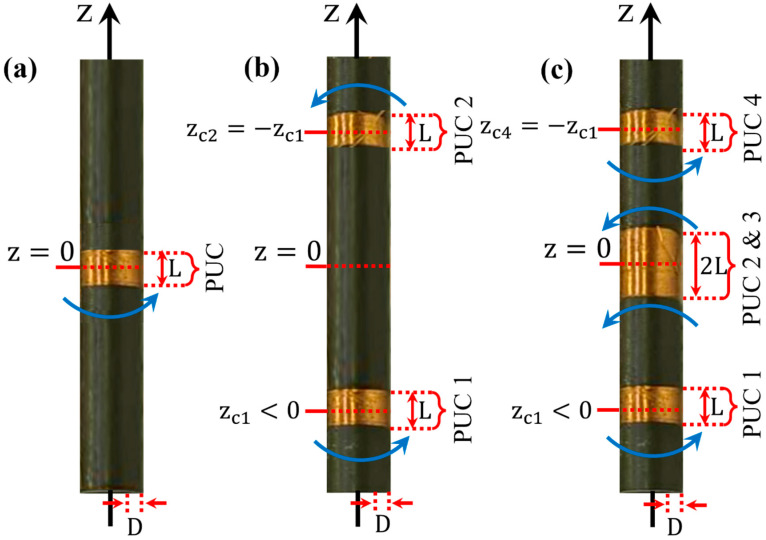
The three representative basic coaxial assemblies of PUCs. (**a**) Single PUC placed with its center at the plane z=0. (**b**) Assembly of two nominally identical, coaxial PUCs, 1 and 2, combined in the FDC. The two PUCs are placed symmetrically at about z=0, $z_{c1}<0$ and $z_{c2}=-z_{c1}>0$, respectively, and have opposite winding directions (see the blue arrows). This ensures that the assembly is not excited by a uniform magnetic field. (**c**) Assembly of four nominally identical, coaxial PUCs, 1, 2, 3 and 4, combined in the SDC. The first, PUC 1, and fourth, PUC 4, are single (outer PUCs) and are placed symmetrically at about z=0, at $z_{c1}<0$ and, $z_{c4}=-z_{c1}>0$, respectively. The middle PUCs, 2 and 3, actually form a double coil that is centered at z=0. The outer PUCs, 1 and 4, have the same winding direction, which is opposite to that of the inner PUCs, 2 and 3 (see the blue arrows). This ensures that the assembly is excited neither by a uniform or linearly varying magnetic field. (**a**–**c**) In all cases, ZDC, FDC and SDC, the assembly of PUCs is adjusted on an insulating, hollow cylinder with an outer radius, $R_{2}$, and inner radius, $R_{1}$. Each PUC has the same nominal number of turns, N_tot_, length, L, inner/outer radius, $R_{1}$/$R_{2}$, and thickness, $D=R_{2}-R_{1}$, and is wound of insulated thin copper wire of thickness, d. The time-varying moment of a sample, $m\left(t\right)$, placed on the z-axis, imposes a time-varying magnetic flux, $\Phi \left(t\right)$, to the PUCs that, in turn, induces an output AC voltage signal, $V_{AC}(t)$.

Here, we perform detailed and exact mathematical modeling (with only minor assumptions/approximations) of the sensing ability of assemblies of PUCs, aligned coaxially to the z-axis, in the form of an array: the so-called zero-derivative configuration (ZDC; 1 PUC), first-derivative configuration (FDC; 2 PUCs) and second-derivative configuration (SDC; 4 PUCs), as illustrated in Figure 1a–c below. In the literature, these configurations are also termed “gradiometers of zero order”, “first order” and “second order”, respectively (see below). Our mathematical modeling of the sensing ability of all three assemblies is performed for a model case of physics/materials science: we consider the AC magnetic susceptibility (ACMS) of a soft ferromagnetic sample placed along the axis of the assembly (that is, along the z-axis; see Figure 1a–c below) at an arbitrary position, while subjected to an externally applied, harmonic, uniform magnetic field, $H_{ext}\left(r,t\right)=H_{0}\cos\left(\omega t\right)\hat{z}$. For the mathematical description of the magnetic moment, $m\left(t\right)$, of the soft ferromagnetic sample, we employ the concept of an ideal magnetic dipole (MD) due to the following: all kinds of magnetometers, irrespective of their operation principle (vibrating sample magnetometers (VSMs), superconducting quantum interference device magnetometers (SQUIDs), etc.) treat even macroscopic samples as ideal magnetic dipoles. This approximation is employed due to the difficulty in the mathematical treatment when a nonideal MD is considered; any calculations on macroscopic samples for the estimation of their magnetic moment should rely on the integration of their magnetization over the entire volume. This is why, in practice, all magnetometers employ the mathematical approximation of an ideal MD in the fitting procedure performed by their software to estimate the magnetic moment of the studied sample (irrespective of its dimensions). Accordingly, in our case, we employ the concept of an ideal MD to describe the studied sample. The induced time-varying magnetic dipole moment, $m\left(t\right)$, of the sample produces a time-varying magnetic flux, $\Phi \left(t\right)$, to the PUCs, eventually inducing an AC voltage signal, $V_{AC}(t)$. The so-called sensing function quantifies the sensing ability of each assembly and is accessed through closed-form expressions that incorporate all macroscopic characteristics of realistic PUCs, including their dimensions and relative positions [6]. Though focused on physics/materials science (that is, on the ACMS of a soft ferromagnetic sample), the mathematical approach introduced below to describe the sensing function of the studied configurations of PUCs is generic. This makes it useful for many disciplines of science and engineering.

## 2. Mathematical Modeling of Coaxial PUCs in the ZDC, FDC and SDC Assemblies

As mentioned above, the mathematical modeling of the sensing function, $F_{SF}$, ref. [6] is performed for the model case of ACMS of a soft ferromagnetic sample (linear, nonhomogeneous, isotropic). The ACMS is probably the most commonly used technique for investigating the magnetic properties of magnetic and superconducting materials [2,3,4,5,6]. Except for the necessary experimental hardware (cryostat, temperature controller, sample probe, lock-in amplifier, function generator, PC and other peripheral electronics), a homemade ACMS setup is based on a set of complementary coils: the primary coil, which applies the excitation AC magnetic field to the sample; and the secondary coils (the PUCs), which inductively sense the response of the specimen, ultimately providing an AC voltage signal, $V_{AC}(t)$, at their output [6]. Various assemblies of PUCs have been explored so far in the literature [21,22,23,24,25,26,27]. The ones commonly used are based on the coaxial adjustment of one, two and four PUCs in the ZDC, FDC and SDC, as discussed below (in the literature, these configurations are also termed “gradiometers of zero order”, “first order” and “second order, respectively) [21,22,23,24,25,26,27]. The three cases of coaxial assemblies of PUCs, also termed magnetometers, are presented in Figure 1a–c below.

The ZDC refers to the case of a single PUC that is placed with its center at the plane z=0, Figure 1a. The FDC considers two PUCs, which are placed symmetrically with respect to z=0, at planes $z=z_{c1}<0$ (the first) and $z=z_{c2}=-z_{c1}>0$ (the second), Figure 1b. Finally, the SDC refers to the case of four PUCs that are placed symmetrically with respect to z=0, at planes $z=z_{c1}<0$ (the first), $z=z_{c2}<0$ (the second), $z=z_{c3}=-z_{c2}>0$ (the third) and $z=z_{c4}=-z_{c1}>0$ (the fourth), Figure 1c. In all cases investigated here, the PUCs are coaxial; as shown in Figure 1a–c, the assembly is adjusted on an insulating, hollow cylinder with outer radius, R_2_ and inner radius, R_1_. Also, all PUCs have the same nominal number of turns, N_tot_, length, L, inner/outer radius, R_1_/R_2_, and thickness, $D=R_{2}-R_{1}$ and are wound by using insulated thin copper wire of thickness, d. The soft ferromagnetic sample (linear, nonhomogeneous and isotropic) under investigation is placed at the interior, on the axis, of the hollow cylinder. An additional outer coil, with a high length/diameter ratio, the primary coil (not shown in Figure 1a–c) provides the external, harmonic, uniform magnetic field, $H_{ext}\left(r,t\right)=H_{0}\cos\left(\omega t\right)\hat{z}$. In turn, $H_{ext}\left(r,t\right)$, imposes a time-varying magnetization, $M\left(r,t\right)=\chi_{m}\left(r\right)H_{ext}\left(r,t\right)$, to the sample, where $\chi_{m}\left(r\right)$ is its magnetic susceptibility, which, in general, is nonhomogeneous. The sample is represented by an ideal MD of moment $m\left(t\right)=m\left(t\right)\hat{z}=\int_{V}M\left(r,t\right)dV$. In turn, $m\left(t\right)$ will impose a time-varying magnetic flux, $\Phi \left(t\right)$, that eventually induces an AC voltage signal, $V_{AC}(t)$, at the output of the assembly of PUCs.

It should be stressed that the operation of the magnetometer of coaxial PUCs will be successful when the induced AC voltage, $V_{AC}(t)$, stems exclusively from the hosted sample. Thus, the contribution of the triggering cause, that is, of the harmonic, uniform magnetic field, $H_{ext}\left(r,t\right)=H_{0}\cos\left(\omega t\right)\hat{z}$, applied by the primary coil, should be rejected by the PUCs. This is feasible for the cases of FDC and SDC discussed below based on the building block of a single PUC (that is, ZDC) that obviously cannot reject $H_{ext}\left(r,t\right)$. Starting with the scheme of SDC, Figure 1c, the four PUCs are connected in the following reasoning: the two outer single PUCs have the same winding direction but opposite to that of the two inner PUCs. It can be shown that, in this scheme, the voltage induced by both a uniform and a linearly varying harmonic magnetic field, $H_{ext}\left(r,t\right)$, is rejected by the four PUCs (see Appendix A). Thus, in the SDC magnetometer, the only contribution to $V_{AC}(t)$ originates from the moment of the sample, $m\left(t\right)$. Likewise, in the scheme of FDC, Figure 1b, the two PUCs are connected with opposite winding direction so that the voltage induced by a uniform harmonic magnetic field, $H_{ext}\left(r,t\right)$, is rejected (see Appendix B). Thus, in the FDC magnetometer as well, the only contribution to $V_{AC}(t)$ originates from the moment of the sample, $m\left(t\right)$. Finally, referring to the scheme of ZDC, Figure 1a, the single PUC cannot reject the voltage induced by the uniform harmonic magnetic field. This is why this scheme is not used in practice, except when other external means are employed to reject the undesired voltage component (for instance, by using an extra, peripheral cancellation/compensation coil). Nevertheless, the ZDC magnetometer is the starting point of our theoretical modeling, since the single PUC is the building element for the construction of the FDC and SDC assemblies.

Our ultimate goal is to find the sensing function, $F_{SF}$, ref. [6] which quantifies the sensing ability of an assembly of PUCs. Ideally, $F_{SF}$ should be a closed-form expression and should incorporate all macroscopic characteristics (including dimensions and relative positions) of all constituent PUCs of each assembly. Knowing $F_{SF}$ will enable us to specify the optimum characteristics (number of turns, N_tot_, length, L, inner/outer radius, $R_{1}$/$R_{2}$, thickness, $D=R_{2}-R_{1}$ and position of the sample along the z-axis) that maximize $V_{AC}(t)$. Thus, the characteristics of the PUCs can be tailored according to given sensing specifications. In the subsections below, we discuss these issues in detail and provide a closed-form expression for the F_SF_ of each assembly, that is, $F_{PUC-ZDC}$, $F_{PUC-FDC}$ and $F_{PUC-SDC}$, for the PUCs in the ZDC, FDC and SDC, respectively.

### 2.1. One PUC in the ZDC

**Figure 2:**
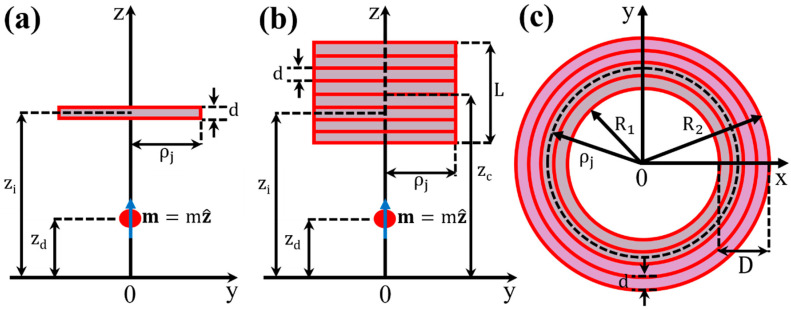
Geometric features of a PUC of 1-turn and of N_tot_ turns, in the ZDC. (**a**) Side view of the PUC of 1-turn of radius, $\rho_{j}$, with its center, $z_{i}$, at the z-axis and its surface parallel to the xy-plane. (**b**) Side view of the PUC of N_tot_ turns. A layer of N turns with length, L, and radius, $\rho_{j}$, with its center at a random position, $z_{c}$, of the z-axis. (**c**) Top view of the PUC of N turns per layer and K layers, so that $N_{tot}=N\cdot K$. Its thickness is, $D=R_{2}-R_{1}=d\cdot K$, where d is the thickness of the wire. In all cases, there is an MD placed at a random position, $z_{d}$, on the z-axis, with its magnetic moment parallel to the z-axis, $m(t)=m(t)\hat{z}$.

The ZDC refers to a single PUC that is placed symmetrically at about z=0, as shown in Figure 1a. This configuration does not include a cancelation/compensation coil. As discussed above, due to this disadvantage, the ZDC has limited use. Nevertheless, it is the absolute building element of any other assembly of PUCs. Due to this fact, here we pay special attention to the mathematical modeling of the single PUC of realistic characteristics, in the presence of a specimen that is described by an ideal MD. To do so, we must first calculate the magnetic flux recorded by a 1-turn PUC of radius, $\rho_{j}$, with its center, $z_{i}$, coinciding with the z-axis, and also, its surface parallel to the xy-plane, as shown in Figure 2a. The MD is placed at the random position, $z_{d}$, on the z-axis with its magnetic moment parallel to the z-axis, $m(t)=m(t)\hat{z}$. The magnetic field originating from an MD of moment, $m(t)=m(t)\hat{z}$, is given by the standard expression ${Β}_{MD}\left(r\right)=(\mu_{0}/4\pi )(3r\left(r\cdot m\left(t\right)\right)-m\left(t\right))/r^{5}$ (for instance, see [28]). Starting from this expression, with relatively easy algebraic calculations, we get the following expression:

(1) $${Β}_{MD}\left(\rho ,z\right)=\frac{\mu_{0}m(t)}{4\pi {\left[\rho^{2}+{\left(z-z_{d}\right)}^{2}\right]}^{5/2}}\left[\rho \left(z-z_{d}\right)\hat{\rho }+\left[-\rho^{2}+2{\left(z-z_{d}\right)}^{2}\right]\hat{z}\right]$$

where ${Β}_{MD}\left(\rho ,z\right)$ (in T: Tesla) is the magnetic field of the MD in cylindrical coordinates and $\mu_{0}$ (in H/m: Henry/meters) is the magnetic permeability of free space. It should be noted that since expression (1) is based on cylindrical coordinates, it facilitates all forthcoming calculations very effectively due to the cylindrical symmetry of the PUCs employed in all ZDC, FDC and SDC studied here.

Integrating over the surface of the PUC of 1-turn results in the magnetic flux [28]

(2) $$\Phi_{1-turn}\left(\rho_{j},z_{i},z_{d}\right)=\int_{0}^{2\pi }\int_{0}^{\rho_{j}}{Β}_{MD}\left(\rho ,z_{i}\right)\cdot da$$

where $da=\rho d\rho d\varphi \hat{z}$ is the surface element of the elementary PUC of 1-turn. Only the z component of the expression (2) contributes to the magnetic flux, $\Phi_{1-turn}\left(\rho_{j},z_{i},z_{d}\right)$, so we have

(3) $$\Phi_{1-turn}\left(\rho_{j},z_{i},z_{d}\right)=\frac{\mu_{0}m(t)}{2}\frac{\rho_{j}^{2}}{{\left(\rho_{j}^{2}+{\left(z_{i}-z_{d}\right)}^{2}\right)}^{3/2}}$$

We now consider a PUC of N_tot_ turns, of length, L, and thickness, $D=R_{2}-R_{1}$, where R_1_ and R_2_ are the inner and outer radius of the PUC, which in the most general case is centered at a random position, $z_{c}$, of the z-axis. The above geometric features of the PUC of N_tot_ turns are shown in Figure 2b,c. The PUC consists of K layers of uniform winding, with each layer consisting of N-turns. Thus, the total number of turns, N_tot_, is given by

(4) $$N_{tot}=N\cdot K$$

The length, L and thickness, D, of the PUC are related to the N turns and K layers, respectively, through the expressions

(5) L=N·d

(6) D=K·d

where d is the thickness of the copper wire. Also, the position, $z_{i}$, and radius, $\rho_{j}$, of each turn of the PUC of N_tot_ turns are given by the expressions

(7) $$z_{i}=z_{1}+\left(i-1\right)d$$

(8) $$\rho_{j}=\rho_{1}+\left(j-1\right)d$$

where i=1,2,…,N number the turns of each layer and j=1,2,…,K number the layers. Thus, the difference of two consecutive, $z_{i}$ and $\rho_{j}$, are given by the expressions

(9) $$\underset{\Delta z_{i}}{\underbrace{z_{i+1}-z_{i}}}=d\overset{\left(5\right)}{\Rightarrow }\Delta z_{i}=\frac{L}{N}$$

(10) $$\underset{\Delta \rho_{j}}{\underbrace{\rho_{j+1}-\rho_{j}}}=d\overset{\left(6\right)}{\Rightarrow }\Delta \rho_{j}=\frac{D}{K}$$

The total magnetic flux recorded by the PUC of N_tot_ turns due to the MD will be the sum of the magnetic fluxes, $\Phi_{1-turn}^{i,j}\left(z_{d}\right)$, of each turn

(11) $$\Phi_{ZDC}^{\;}\left(z_{d}\right)=\sum_{j=1}^{K}\sum_{i=1}^{N}\Phi_{1-turn}\left(\rho_{j},z_{i},z_{d}\right)\overset{(3)\;}{\Rightarrow }\Phi_{ZDC}^{\;}\left(z_{d}\right)=\frac{\mu_{0}m(t)}{2}\sum_{j=1}^{K}\sum_{i=1}^{N}\frac{\rho_{j}^{2}}{{\left(\rho_{j}^{2}+{\left(z_{i}-z_{d}\right)}^{2}\right)}^{3/2}}$$

From the combination of the expressions (4), (9) and (10), it follows that $\frac{N_{tot}}{LD}\Delta z_{i}\Delta \rho_{j}=1$, thus the expression (11) becomes

(12) $$\Phi_{ZDC}^{\;}\left(z_{d}\right)=\frac{N_{tot}}{LD}\frac{\mu_{0}m(t)}{2}\sum_{j=1}^{K}\sum_{i=1}^{N}\underset{f\left(z_{i}\right)}{\underbrace{\frac{\rho_{j}^{2}}{{\left(\rho_{j}^{2}+{\left(z_{i}-z_{d}\right)}^{2}\right)}^{3/2}}}}\Delta z_{i}\Delta \rho_{j}$$

**Figure 3:**
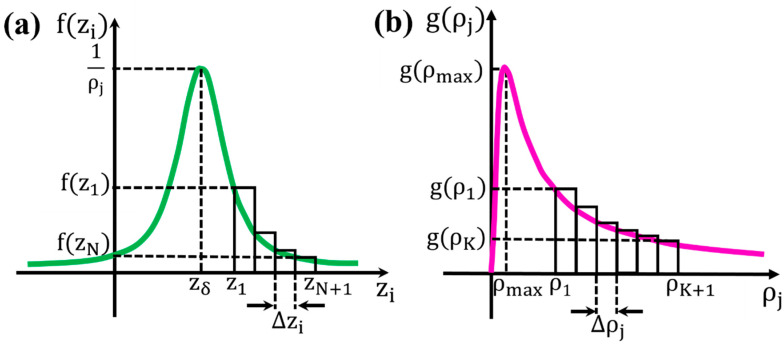
(**a**) Plot of the function $f\left(z_{i}\right)$ and the N orthogonal parallelograms that approximate the curve. (**b**) Plot of the function $g\left(\rho_{j}\right)$ and the K orthogonal parallelograms that approximate the curve. In both cases, the thickness of the orthogonal parallelograms, $\Delta z_{i}$ and $\Delta \rho_{j}$, is the same as the thickness of the copper wire, d.

As we can see from expression (12) and Figure 3a, the calculation of the sum with respect to i is reduced to the calculation of N rectangular parallelograms with height, $f(z_{i})$, and width, $\Delta z_{i}$. Due to the small thickness of the copper wire, $d=\Delta z_{i}$, we can make the approximation

$$\sum_{i=1}^{N}f\left(z_{i}\right)\Delta z_{i}≃\int_{z_{1}}^{z_{N}}f\left(z\right)dz$$

The integration over z is given by

$$\int \frac{\rho_{j}^{2}}{{\left(\rho_{j}^{2}+{\left(z-z_{d}\right)}^{2}\right)}^{3/2}}dz=\frac{z-z_{d}}{\sqrt{\rho_{j}^{2}+{\left(z-z_{d}\right)}^{2}}}+C$$

Thus, the magnetic flux, $\Phi_{ZDC}^{\;}\left(z_{d}\right)$, becomes

(13) $$\Phi_{ZDC}^{\;}\left(z_{d}\right)=\frac{N_{tot}}{LD}\frac{\mu_{0}m(t)}{2}\sum_{j=1}^{K}\underset{g\left(\rho_{j}\right)}{\underbrace{\left[\frac{z_{c}+\frac{L}{2}-z_{d}}{\sqrt{\rho_{j}^{2}+{\left(z_{c}+\frac{L}{2}-z_{d}\right)}^{2}}}-\frac{z_{c}-\frac{L}{2}-z_{d}}{\sqrt{\rho_{j}^{2}+{\left(z_{c}-\frac{L}{2}-z_{d}\right)}^{2}}}\right]}}\Delta \rho_{j}$$

We continue now we the calculation of the sum with respect to j. From expression (13) and Figure 3b, the calculation of the sum with respect to j is reduced to the calculation of K rectangular parallelograms with height, $g(\rho_{j})$, and width, $\Delta \rho_{j}$. Due to the small thickness of the copper wire, $d=\Delta \rho_{j}$, we can make the following approximation:

$$\sum_{j=1}^{K}g(\rho_{j})\Delta \rho_{j}≃\int_{\rho_{1}}^{\rho_{K}}g\left(\rho \right)d\rho$$

The integration over ρ is given by

$$\int \frac{z-z_{d}}{\sqrt{\rho^{2}+{\left(z-z_{d}\right)}^{2}}}d\rho =\left|z-z_{d}\right|\ln\left[\frac{\rho }{z-z_{d}}+\sqrt{1+{\left(\frac{\rho }{z-z_{d}}\right)}^{2}}\right]+C$$

Finally, the recorded magnetic flux by the PUC of N_tot_ turns is given by the expression

(14) $$\Phi_{ZDC}^{\;}\left(z_{d}\right)=\frac{N_{tot}}{LD}\frac{\mu_{0}m(t)}{2}\left[\left|z_{c}+\frac{L}{2}-z_{d}\right|A^{+}\left(z_{d}\right)\left.-\left|z_{c}-\frac{L}{2}-z_{d}\right|A^{-}\left(z_{d}\right)\right]\right.$$

where $A^{+/-}\left(z_{d}\right)$ is given by

(15) $$A^{+/-}\left(z_{d}\right)=\ln\left[\frac{R_{2}sgn\left(z_{c}\pm \frac{L}{2}-z_{d}\right)+\sqrt{R_{2}^{2}+{\left(z_{c}\pm \frac{L}{2}-z_{d}\right)}^{2}}}{R_{1}sgn\left(z_{c}\pm \frac{L}{2}-z_{d}\right)+\sqrt{R_{1}^{2}+{\left(z_{c}\pm \frac{L}{2}-z_{d}\right)}^{2}}}\right]$$

**Figure 4:**
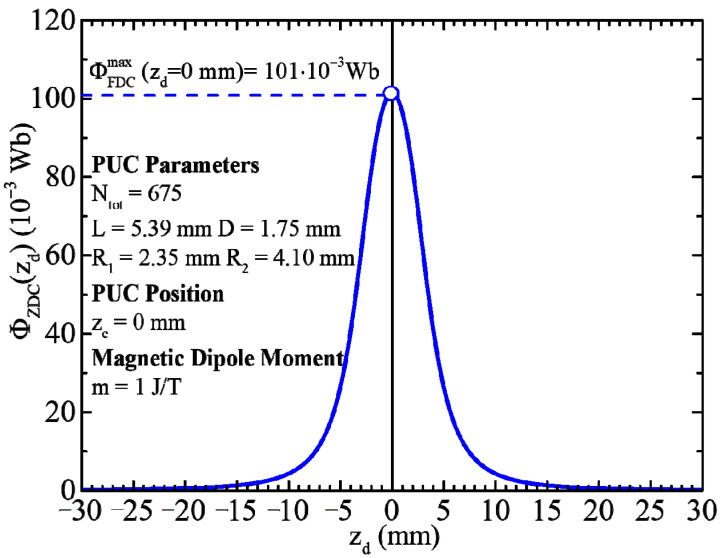
Simulation of the recorded magnetic flux of a ZDC, $\Phi_{ZDC}$, with the realistic parameters $N_{tot}=675$, $R_{1}=2.35\;mm$, $R_{2}=4.10\;mm$, L=5.39 mm, D=1.75 mm and $z_{c}=0\;mm$ as a function of an MD position, $z_{d}$, with magnetic dipole moment, m=1 J/T.

Simulations of the expression (14) were performed by using the realistic parameters $N_{tot}=675$, $R_{1}=2.35\;mm$, $R_{2}=4.10\;mm$, L=5.39 mm, D=1.75 mm, $z_{c}=0\;mm$ and m=1 J/T. These parameters were employed because they refer to the actual case met in relevant magnetometers used in experimental practice. For instance, in our laboratory, we have PUCs (ZDC, FDC and SDC) having parameters ($N_{tot}$, $R_{1}$, $R_{2}$, L, D and $z_{c}$) in the range of the above ones (see Figure 1a–c) and [6]). These simulations evidence that the recorded magnetic flux from the ZDC reaches its maximum when the MD is placed at the center of the PUC, as shown in Figure 4. To do this calculation, we must substitute $z_{d}=z_{c}$ in the expressions (14) and (15) and use the identity $\ln\left(\sqrt{1+x^{2}}+x\right)=-\ln\left(\sqrt{1+x^{2}}-x\right)$

(16) $$\Phi_{ZDC}^{max}=\frac{N_{tot}}{2D}\mu_{0}m(t)\ln\left[\frac{R_{2}+\sqrt{R_{2}^{2}+{\left(\frac{L}{2}\right)}^{2}}}{R_{1}+\sqrt{R_{1}^{2}+{\left(\frac{L}{2}\right)}^{2}}}\right]$$

In an AC magnetic susceptibility experiment where the MD is positioned at the center of the PUC of N_tot_ turns, the maximum value of the inductive voltage of the ZDC, $V_{AC-ZDC}^{max}\left(t\right)$, according to Faraday’s law, $V\left(t\right)=-d\Phi \left(t\right)/dt$, and the expression (16) is given by

(17) $$V_{AC-ZDC}^{max}\left(t\right)=-\frac{d\Phi_{ZDC}^{max}\left(t\right)}{dt}=-\mu_{0}\frac{dm\left(t\right)}{dt}\;F_{PUC-ZDC}$$

where $F_{PUC-ZDC}=F_{SF}$, the sensing function [6] of the ZDC assembly:

(18) $$F_{PUC-ZDC}=F_{SF}=\frac{N_{tot}}{2D}\ln\left[\frac{R_{2}+\sqrt{R_{2}^{2}+{\left(\frac{L}{2}\right)}^{2}}}{R_{1}+\sqrt{R_{1}^{2}+{\left(\frac{L}{2}\right)}^{2}}}\right]$$

The expression (18) quantifies the sensing ability of a PUC using all of each macroscopic characteristics (number of turns, N_tot_, length, L, inner/outer radius, $R_{1}$/$R_{2}$ and thickness, $D=R_{2}-R_{1}$).

### 2.2. Two Coaxial PUCs in the FDC

The FDC refers to two coaxial PUCs, each one of N_tot_ turns, that are placed symmetrically at about z=0, with its centers at $z_{c1}<0$ (the first) and $z_{c2}=-z_{c1}>0$ (the second), as shown in Figure 1b. The two PUCs have windings of opposite directions. Thus, the magnetic flux recorded by the FDC due to the MD is obtained by using the results of the previous section:

(19) $$\Phi_{FDC}\left(z_{d}\right)=\Phi_{ZDC}^{1}\left(z_{d}\right)-\Phi_{ZDC}^{2}\left(z_{d}\right)$$

where $\Phi_{ZDC}^{1}\left(z_{d}\right)$ and $\Phi_{ZDC}^{2}\left(z_{d}\right)$ are the magnetic flux being recorded by the PUC 1 and 2, respectively. The negative sign is due to the opposite winding of the PUCs. Also, notice that the subscript ZDC in each term indicates that the two PUCs are independent/noninteracting so that expression (14) of Section 2.1 applies for each one of them; the entire magnetic flux is simply the superposition of the ones recorded independently by the two PUCs. Thus, using the expressions (14) and (15) and the fact that $z_{c2}=-z_{c1}>0$, expression (19) becomes

(20) $$\begin{array}{c} \Phi_{FDC}\left(z_{d}\right)=\frac{N_{tot}}{LD}\frac{\mu_{0}m(t)}{2}\left[\left|z_{c2}-\frac{L}{2}+z_{d}\right|A_{1}^{+}\left(z_{d}\right)-\left|z_{c2}+\frac{L}{2}+z_{d}\right|A_{1}^{-}\right.\left(z_{d}\right)\\ \left.-\left(\left|z_{c2}+\frac{L}{2}-z_{d}\right|A_{2}^{+}\left(z_{d}\right)-\left|z_{c2}-\frac{L}{2}-z_{d}\right|A_{2}^{-}\left(z_{d}\right)\right)\right] \end{array}$$

where $A_{1}^{+/-}\left(z_{d}\right)$ and $A_{2}^{+/-}\left(z_{d}\right)$ are given by:

(21) $$A_{1}^{+/-}\left(z_{d}\right)=\ln\left[\frac{-R_{2}sgn\left(z_{c2}\mp \frac{L}{2}+z_{d}\right)+\sqrt{R_{2}^{2}+{\left(z_{c2}\mp \frac{L}{2}+z_{d}\right)}^{2}}}{-R_{1}sgn\left(z_{c2}\mp \frac{L}{2}+z_{d}\right)+\sqrt{R_{1}^{2}+{\left(z_{c2}\mp \frac{L}{2}+z_{d}\right)}^{2}}}\right]$$

(22) $$A_{2}^{+/-}\left(z_{d}\right)=\ln\left[\frac{R_{2}sgn\left(z_{c2}\pm \frac{L}{2}-z_{d}\right)+\sqrt{R_{2}^{2}+{\left(z_{c2}\pm \frac{L}{2}-z_{d}\right)}^{2}}}{R_{1}sgn\left(z_{c2}\pm \frac{L}{2}-z_{d}\right)+\sqrt{R_{1}^{2}+{\left(z_{c2}\pm \frac{L}{2}-z_{d}\right)}^{2}}}\right]$$

**Figure 5:**
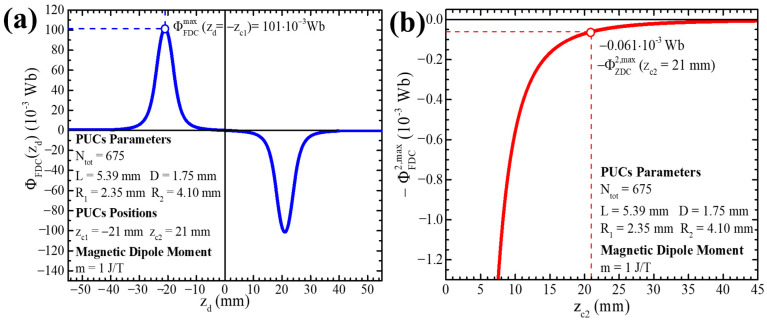
(**a**) Simulation of the recorded magnetic flux of a FDC, $\Phi_{FDC}$, which is centered at the origin of the z-axis, as a function of an MD position, $z_{d}$. (**b**) Simulation of the contribution of the cancelation/compensation PUC 2, ${-\Phi }_{ZDC}^{2,max}$, as a function of the center of the PUC 2, $z_{c2}$. For both simulations we used the realistic parameters $N_{tot}=675$, $R_{1}=2.35\;mm$, $R_{2}=4.10\;mm$, L=5.39 mm, D=1.75 mm, $z_{c2}=21\;mm$ and m=1 J/T.

By simulating expression (20) for the realistic parameters $N_{tot}=675$, $R_{1}=2.35\;mm$, $R_{2}=4.10\;mm$, L=5.39 mm, D=1.75 mm, $z_{c2}=21\;mm$ and m=1 J/T, we easily see that the recorded magnetic flux from the FDC reaches its maximum when the MD is placed at the center of one of the PUCs, as shown in Figure 5a. Accordingly, let us fix the position of the MD at the center of PUC 1. The maximum can then be calculated by substituting $z_{d}=z_{c1}=-z_{c2}$ in the expressions (20)–(22) and using the identity $\ln\left(\sqrt{1+x^{2}}+x\right)=-\ln\left(\sqrt{1+x^{2}}-x\right)$, we get

(23) $$\begin{array}{c} \Phi_{FDC}^{max}=\frac{N_{tot}}{LD}\frac{\mu_{0}m(t)}{2}\left[L\ln\left[\frac{R_{2}+\sqrt{R_{2}^{2}+{\left(\frac{L}{2}\right)}^{2}}}{R_{1}+\sqrt{R_{1}^{2}+{\left(\frac{L}{2}\right)}^{2}}}\right]-\left(\left(2z_{c2}+\frac{L}{2}\right)\ln\left[\frac{R_{2}+\sqrt{R_{2}^{2}+{\left(2z_{c2}+\frac{L}{2}\right)}^{2}}}{R_{1}+\sqrt{R_{1}^{2}+{\left(2z_{c2}+\frac{L}{2}\right)}^{2}}}\right]\right.\right.\\ \left.\left.-\left(2z_{c2}-\frac{L}{2}\right)\ln\left[\frac{R_{2}+\sqrt{R_{2}^{2}+{\left(2z_{c2}-\frac{L}{2}\right)}^{2}}}{R_{1}+\sqrt{R_{1}^{2}+{\left(2z_{c2}-\frac{L}{2}\right)}^{2}}}\right]\right)\right] \end{array}$$

The first term of expression (23) is the contribution of PUC 1 (i.e., the PUC with the MD at its center, $\Phi_{ZDC}^{1,max}$), while the last two terms are the contribution of the PUC 2, $\Phi_{ZDC}^{2,max}$. From those two last terms, by using a quantitative criterion, we can estimate the distance at which the signal of the MD has negligible contribution to one of the PUCs. As shown in Figure 5b, we notice that as we increase the distance between the two PUCs (i.e., as $z_{c2}$ increases, the contribution of the PUC 2 becomes negligible. This is expected, since PUC 2 is very far away from the MD, which is placed at the center of PUC 1.

Thus, we can make the approximation $\Phi_{ZDC}^{2,max}≃0$, so the expression (23) that gives the maximum magnetic flux of the FDC becomes

(24) $$\Phi_{FDC}^{max}≃\Phi_{ZDC}^{1,max}=\frac{N_{tot}}{2D}\mu_{0}m(t)\ln\left[\frac{R_{2}+\sqrt{R_{2}^{2}+{\left(\frac{L}{2}\right)}^{2}}}{R_{1}+\sqrt{R_{1}^{2}+{\left(\frac{L}{2}\right)}^{2}}}\right]$$

The quantitative adequacy of expression (24) is confirmed through the direct comparison of expression (23) with expression (16) by means of simulations using standard software (Origin 8.5). According to the realistic parameters that we used for the simulations, the difference between the expressions (16) and (23) is on the order of

$$\left|\frac{\Phi_{FDC}^{max}-\Phi_{ZDC}^{1,max}}{\Phi_{FDC}^{max}}\;\right|100\;\%=\left|\frac{\Phi_{ZDC}^{2,max}}{\Phi_{FDC}^{max}}\right|100\;\%=\left|\frac{-0.061\cdot 10^{-3}Wb}{101\cdot 10^{-3}Wb}\right|100\;\%=0.06\;\%$$

From the expression (24), we easily see that the induced AC voltage of the FDC assembly, $V_{AC-FDC}^{max}(t)$, reaches its maximum when the MD is placed at the center of one of the PUCs:

(25) $$V_{AC-FDC}^{max}(t)=-\frac{d\Phi_{FDC}^{max}\left(t\right)}{dt}=-\mu_{0}\frac{dm\left(t\right)}{dt}F_{PUC-FDC}$$

with $F_{PUC-FDC}$, the sensing function [6] for the case of the FDC:

(26) $$F_{PUC-FDC}=F_{SF}=\frac{N_{tot}}{2D}\ln\left[\frac{R_{2}+\sqrt{R_{2}^{2}+{\left(\frac{L}{2}\right)}^{2}}}{R_{1}+\sqrt{R_{1}^{2}+{\left(\frac{L}{2}\right)}^{2}}}\right]$$

Then, the other PUC of N_tot_ turns actually plays the role of the cancelation/compensation coil that rejects the voltage induced by the uniform harmonic magnetic field, $H_{ext}\left(r,t\right)=H_{0}\cos\left(\omega t\right)\hat{z}$, to the first one, which hosts the MD. Finally, the result obtained above for the case of FDC is very useful for the case of geophysics, aerospace physics, etc., where the triggering cause originates from the exterior of the assembly of PUCs, especially in cases where a uniform magnetic field should be excluded (see Appendix B).

### 2.3. Four PUCs in the SDC

The SDC refers to four coaxial PUCs of N_tot_ turns that are placed symmetrically at about z=0, with their centers at $z_{c1}<0$ (the first), $z_{c2}<0$ (the second), $z_{c3}=-z_{c2}>0$ (the third) and $z_{c4}=-z_{c1}>0\;($the fourth). Furthermore, the lower surface, $z_{c3}-L/2$, of PUC 3 coincides with the upper surface, $z_{c2}+L/2$, of PUC 2 at z=0, creating in this way a double PUC of 2N_tot_ turns, as shown in Figure 1c. Referring to their connection, the two outer single PUCs (1 and 4 in Figure 1c) have the same winding direction but opposite to that of the two inner PUCs (2 and 3 in Figure 1c). Thus, the magnetic flux recorded by the SDC due to the MD is given by the expression

(27) $$\Phi_{SDC}\left(z_{d}\right)=-\Phi_{ZDC}^{1}\left(z_{d}\right)+\Phi_{ZDC}^{2}\left(z_{d}\right)+\Phi_{ZDC}^{3}\left(z_{d}\right)-\Phi_{ZDC}^{4}\left(z_{d}\right)$$

where $\Phi_{ZDC}^{i}\left(z_{d}\right)$, with i=1,2,3,4, are the magnetic fluxes being recorded by the corresponding PUC of N_tot_ turns. The negative sign is due to the opposite winding direction, as described above. We recall that the subscript ZDC in each term indicates that the four PUCs are independent/noninteracting so that expression (14) of Section 2.1 applies for each one of them; the entire magnetic flux is simply the superposition of the ones recorded independently by the four PUCs. Thus, using the expressions (14) and (15) and the fact that $z_{c3}=-z_{c2}$ and $z_{c4}=-z_{c1},$ the expression (27) becomes

(28) $$\begin{array}{c} \Phi_{SDC}\left(z_{d}\right)=\frac{N_{tot}}{LD}\frac{\mu_{0}m(t)}{2}\left[-\left(\left|z_{c4}-\frac{L}{2}+z_{d}\right|A_{1}^{+}\left(z_{d}\right)-\left|z_{c4}+\frac{L}{2}+z_{d}\right|A_{1}^{-}\left(z_{d}\right)\right)+\left.\left|L-z_{d}\right|A_{3}^{+}\right.\right.\left(z_{d}\right)\\ \left.-\left.\left|L+z_{d}\right|A_{2}^{-}\left(z_{d}\right)\right.-\left(\left|z_{c4}+\frac{L}{2}-z_{d}\right|A_{4}^{+}\left(z_{d}\right)-\left|z_{c4}-\frac{L}{2}-z_{d}\right|A_{4}^{-}\left(z_{d}\right)\right)\right]\;\; \end{array}$$

where $A_{1}^{+/-}\left(z_{d}\right)$, $A_{2}^{-}\left(z_{d}\right)$, $A_{3}^{+}\left(z_{d}\right)$ and, $A_{4}^{+/-}\left(z_{d}\right)$ are given by

(29) $$A_{1}^{+/-}\left(z_{d}\right)=\ln\left[\frac{-R_{2}sgn\left(z_{c4}\mp \frac{L}{2}+z_{d}\right)+\sqrt{R_{2}^{2}+{\left(z_{c4}\mp \frac{L}{2}+z_{d}\right)}^{2}}}{-R_{1}sgn\left(z_{c4}\mp \frac{L}{2}+z_{d}\right)+\sqrt{R_{1}^{2}+{\left(z_{c4}\mp \frac{L}{2}+z_{d}\right)}^{2}}}\right]$$

(30) $$A_{4}^{+/-}\left(z_{d}\right)=\ln\left[\frac{R_{2}sgn\left(z_{c4}\pm \frac{L}{2}-z_{d}\right)+\sqrt{R_{2}^{2}+{\left(z_{c4}\pm \frac{L}{2}-z_{d}\right)}^{2}}}{R_{1}sgn\left(z_{c4}\pm \frac{L}{2}-z_{d}\right)+\sqrt{R_{1}^{2}+{\left(z_{c4}\pm \frac{L}{2}-z_{d}\right)}^{2}}}\right]$$

(31) $$A_{2}^{-}\left(z_{d}\right)=\ln\left[\frac{-R_{2}sgn\left(L+z_{d}\right)+\sqrt{R_{2}^{2}+{\left(L+z_{d}\right)}^{2}}}{-R_{1}sgn\left(L+z_{d}\right)+\sqrt{R_{1}^{2}+{\left(L+z_{d}\right)}^{2}}}\right]$$

(32) $$A_{3}^{+}\left(z_{d}\right)=\ln\left[\frac{R_{2}sgn\left(L-z_{d}\right)+\sqrt{R_{2}^{2}+{\left(L-z_{d}\right)}^{2}}}{R_{1}sgn\left(L-z_{d}\right)+\sqrt{R_{1}^{2}+{\left(L-z_{d}\right)}^{2}}}\right]$$

**Figure 6:**
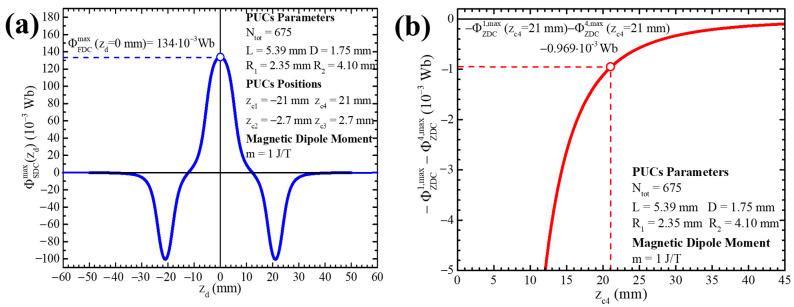
(**a**) Simulation of the recorded magnetic flux of an SDC, $\Phi_{SDC}$, which is centered at the origin of the z-axis, as a function of an MD position, $z_{d}$. (**b**) Simulation of the contribution of the cancelation/compensation PUCs 1 and 4, ${-\Phi }_{ZDC}^{1,max}{-\Phi }_{ZDC}^{4,max}$, as a function of the center of the PUC 4, $z_{c4}$. For both simulations, we used the realistic parameters $N_{tot}=675$, $R_{1}=2.35\;mm$, $R_{2}=4.10\;mm$, L=5.39 mm, D=1.75 mm, $z_{c3}=2.7\;mm$, $z_{c4}=21\;mm$ and m=1 J/T.

By simulating the expression (28) for the realistic parameters $N_{tot}=675$, $R_{1}=2.35\;mm$, $R_{2}=4.10\;mm$, L=5.39 mm, D=1.75 mm, $z_{c3}=2.7\;mm$, $z_{c4}=21\;mm$ and m=1 J/T, we easily see that the recorded magnetic flux from the SDC reaches its maximum when the MD is placed at z=0, which is the center of the double PUC of 2N_tot_ turns, as shown in Figure 6a. To calculate the maximum, we must substitute $z_{d}=0$ in the expressions (28)–(32) and use the identity $\ln\left(\sqrt{1+x^{2}}+x\right)=-\ln\left(\sqrt{1+x^{2}}-x\right):$

(33) $$\begin{array}{c} \Phi_{SDC}^{max}=\frac{N_{tot}}{LD}\mu_{0}m(t)\left[L\ln\left[\frac{R_{2}+\sqrt{R_{2}^{2}+L^{2}}}{R_{1}+\sqrt{R_{1}^{2}+L^{2}}}\right]-\left.\left(z_{c4}+\frac{L}{2}\right)\ln\left[\frac{R_{2}+\sqrt{R_{2}^{2}+{\left(z_{c4}+\frac{L}{2}\right)}^{2}}}{R_{1}+\sqrt{R_{1}^{2}+{\left(z_{c4}+\frac{L}{2}\right)}^{2}}}\right]\right.\right.\\ \left.\left.+\left(z_{c4}-\frac{L}{2}\right)\ln\left[\frac{R_{2}+\sqrt{R_{2}^{2}+{\left(z_{c4}-\frac{L}{2}\right)}^{2}}}{R_{1}+\sqrt{R_{1}^{2}+{\left(z_{c4}-\frac{L}{2}\right)}^{2}}}\right]\right.\right] \end{array}$$

The first term of the expression (33) is the contribution of PUCs 2 and 3 (i.e., the double PUC of 2N_tot_ turns with the MD at its center, $\Phi_{ZDC}^{2,max}+\Phi_{ZDC}^{3,max}$), while the last two terms are the contribution of the single PUCs 1 and 4 of N_tot_ turns each, $-\Phi_{ZDC}^{1,max}-\Phi_{ZDC}^{4,max}$. From those two last terms, through a quantitative criterion, we can estimate the distance in which the signal of the single PUCs 1 and 4 does not affect that of the double PUC 2 and 3. As shown in Figure 6b, we notice that as we increase the distance between each single PUC 1 and 4, with the double PUC (i.e., as $z_{c4}$ increases, the contribution of the single PUCs 1 and 4 becomes negligible). This is expected, since the single PUCs 1 and 4 are far away from the MD, which is at the center of the double PUC 2 and 3. Thus, we can make the approximation ${-\Phi }_{ZDC}^{1,max}-\Phi_{ZDC}^{4,max}≃0$, so the expression (33) that gives the maximum magnetic flux of the SDC becomes

(34) $$\Phi_{SDC}^{max}≃\Phi_{ZDC}^{2,max}+\Phi_{ZDC}^{3,max}=\frac{N_{tot}}{D}\mu_{0}m(t)\ln\left[\frac{R_{2}+\sqrt{R_{2}^{2}+L^{2}}}{R_{1}+\sqrt{R_{1}^{2}+L^{2}}}\right]$$

By comparing the above expression (34) with expressions (16) and (24) of the ZDC and FDC, respectively, we see that the multiplying factor $N_{tot}/2D$ of (16) and (24) has been replaced by $N_{tot}/D$ in (34), since it was originally ${2N}_{tot}/2D$ (the number of turns of the double PUC is twice that of each single PUC). Thus, in expression (34), N_tot_ still refers to the number of turns of each single PUC. In addition, the factor (L/2) that appears in the square root of expressions (16) and (24), has been replaced by L in (34), since the length of the double PUC is twice that of each single PUC.

The quantitative adequacy of expression (34) is confirmed through the direct comparison of expression (33) with expression (16) by means of simulations using standard software (Origin 8.5). According to the realistic parameters that we used for the simulations, the difference between the expressions (16) and (33) is on the order of

$$\left|\frac{\Phi_{SDC}^{max}-\Phi_{ZDC}^{2,max}-\Phi_{ZDC}^{3,max}}{\Phi_{SDC}^{max}}\;\right|100\;\%=\left|\frac{-\Phi_{ZDC}^{1,max}-\Phi_{ZDC}^{4,max}}{\Phi_{SDC}^{max}}\right|100\;\%\;=\left|\frac{-0.969\cdot 10^{-3}Wb}{134\cdot 10^{-3}Wb}\right|100\;\%=0.72\;\%$$

From expression (34), we easily see that the induced AC voltage of the SDC assembly, $V_{AC-SDC}^{max}(t)$, reaches its maximum when the MD is placed at the center of the inner double PUC; that is, at z=0:

(35) $$V_{AC-SDC}^{max}(t)=-\frac{d\Phi_{SDC}^{max}\left(t\right)}{dt}=-\mu_{0}\frac{dm\left(t\right)}{dt}F_{PUC-SDC}$$

with $F_{PUC-SDC}$, the sensing function [6] for the case of the SDC:

(36) $${F_{PUC-SDC}=F}_{SF}=\frac{N_{tot}}{D}\ln\left[\frac{R_{2}+\sqrt{R_{2}^{2}+L^{2}}}{R_{1}+\sqrt{R_{1}^{2}+L^{2}}}\right]$$

The two outer PUCs then actually play the role of the cancelation/compensation coil for each of the inner two PUCs. The result obtained above for the case of SDC is very useful for the case of geophysics, aerospace physics, etc., where the triggering cause originates from the exterior of the assembly of PUCs, especially in cases where both a uniform and linearly varying magnetic field should be excluded (see Appendix A).

By comparing the above expression (36) with expressions (18) and (26) of the ZDC and FDC, respectively, we see that the multiplying factor $N_{tot}/2D$ of (18) and (26) has been replaced by $N_{tot}/D$ in (36), since it was originally ${2N}_{tot}/2D$ (the number of turns of the double PUC is twice that of each single PUC). Thus, in expression (36), N_tot_ still refers to the number of turns of each single PUC. In addition, the factor (L/2) that appears in the square root of expressions (18) and (26) has been replaced by L in (36), since the length of the double PUC is twice that of each single PUC.

### 2.4. Perspectives and Limitations

We close our work with a brief discussion of the perspectives and limitations of the detailed analytical approach reported here. Magnetic field sensors based on conventional PUCs have many advantages over other solid-state sensing units, such as Hall, Giant Magnetoresistance and Tunnel Magnetoresistance, to name just a few [29]. Obviously, the PUCs-based inductive sensors can be easily fabricated and are flexibly adjustable to the needs of every experiment at a practically negligible cost.

This is why they have been used in a wide range of experimental studies on the properties of magnetic and superconducting materials, either in the form of a single PUC or two PUCs, with dimensions adjusted to the size of each sample [30,31,32,33,34,35]. Also, 3D magnetic field sensors based on planar PUCs that can be fabricated relatively easily have been studied by simulations and numerical analyses [36]; single PUC magnetic field sensors, without and with a core for plasma-based and power-cable applications have been reported so far [37,38], and single PUCs have been incorporated in LC resonators for the nondestructive evaluation of materials [39].

Obviously, the detailed analytical approach and the respective closed-form relations of the sensing function reported here for the ZDC, FDC and SDC can be of direct use for the description of scalar arrays of coaxial PUCs having a circular cross-section; that is, of cylindrical PUCs placed in series along an axis. However, more complex vector arrays of noncoaxial, cylindrical PUCs or of coaxial PUCs that have orthogonal cross-sections cannot be treated easily by the analytical procedure reported here. Clearly, such cases that are more complex need a computational approach.

## 3. Conclusions

We performed detailed mathematical modeling by making only minor assumptions/approximations of the magnetic flux-to-voltage transformation ability for three basic assemblies of PUCs aligned coaxially to the z-axis: the ZDC (1 PUC), FDC (2 PUCs) and SDC (4 PUCs). The model case considered here was the time-varying moment of a soft ferromagnetic sample represented by an MD that is placed on the z-axis. For each case, we obtained closed-form expressions of the magnetic flux, $\Phi \left(t\right)$, recorded by the PUCs, of the respective AC voltage signal, $V_{AC}(t)$, induced at their output and of the sensing function, $F_{SF}$, that quantifies the magnetic flux-to-voltage transformation ability of each assembly. All closed-form expressions, $\Phi \left(t\right)$, $V_{AC}(t)$ and $F_{SF}$, incorporate all macroscopic characteristics of realistic PUCs, including their dimensions and relative positions. Though focused on a model case of physics/materials science, the mathematical approach introduced here is both complete and versatile and can be adjusted to describe other relevant configurations of coaxial PUCs, including radically different kinds of external triggering. Also, it can be used to describe more general cases where the sample is placed outside the z-axis, however at the cost of more complicated mathematics. Finally, our generic mathematical approach paves the way for the design and manufacturing of coils with tailored sensing specifications; thus, apart from physics, it can be useful in other disciplines of science and engineering.

## Data Availability

The data that support the findings of this study are available from the corresponding author upon reasonable request.

## Appendix A

Here we prove that the SDC of the four coaxial PUCs rejects the signal that originates from a uniform and a linearly varying magnetic field. The magnetic flux recorded by the SDC in the presence of an externally applied uniform and linearly varying harmonic magnetic field, $B_{ext}\left(z,t\right)=\left(B_{x}\hat{x}+B_{y}\hat{y}+B_{z}z\hat{z}\right)\sin\left(\omega t\right)$, where $B_{x}$, $B_{y}$ and $B_{z}$ are constants, is given by the expression

(A1) $$\Phi_{SDC}\left(t\right)=-\Phi_{ZDC}^{1}\left(t\right)+\Phi_{ZDC}^{2}\left(t\right)+\Phi_{ZDC}^{3}\left(t\right)-\Phi_{ZDC}^{4}\left(t\right)$$

where $\Phi_{ZDC}^{i}\left(t\right)$, with i=1,2,3,4, being the magnetic fluxes being recorded by the respective PUC. The negative sign is due to the opposite winding direction, as described in Section 2.3. The geometric characteristics are shown in Figure 1c for the SDC and in Figure 1a and Figure 2b,c for the ZDC. In order to calculate the magnetic fluxes, $\Phi_{ZDC}^{i}\left(t\right)$, we must first calculate the magnetic flux recorded by each individual 1-turn of the PUCs 1, 2, 3 and 4, $\Phi_{1-turn}^{i}\left(\rho_{j},z_{i},t\right)$, where $z_{i}$ and $\rho_{j}$ are its position and radius, respectively. Only the z component of the $B_{ext}\left(z,t\right)$ contributes to the magnetic flux, $\Phi_{1-turn}^{i}\left(\rho_{j},z_{i},t\right)$; thus,

(A2) $$\Phi_{1-turn}^{i}\left(\rho_{j},z_{i},t\right)=∯B_{ext}\left(z_{i},t\right)\cdot da=\int_{0}^{2\pi }\int_{0}^{\rho_{j}}B_{z}z_{i}\sin\left(\omega t\right)\rho d\rho d\varphi =\pi B_{z}z_{i}\sin\left(\omega t\right)\rho_{j}^{2}$$

The total magnetic flux recorded by the PUCs 1, 2, 3 and 4 due to the $B_{ext}\left(t\right)$ will be the sum of the magnetic fluxes, $\Phi_{1-PUC}^{i}\left(\rho_{j},z_{i},t\right)$, of each turn:

(A3) $$\Phi_{ZDC}^{i}\left(t\right)=\sum_{j=1}^{K}\sum_{i=1}^{N}\Phi_{1-PUC}^{i}\left(\rho_{j},z_{i},t\right)=\pi B_{z}\sin\left(\omega t\right)\sum_{j=1}^{K}\sum_{i=1}^{N}{z_{i}\rho }_{j}^{2}$$

From the combination of the expressions (4), (9) and (10), which consist of the mathematical modeling of the ZDC, it follows that $\frac{N_{tot}}{LD}\Delta z_{i}\Delta \rho_{j}=1$; thus, the expression (A3) becomes

(A4) $$\Phi_{ZDC}^{i}\left(t\right)=\frac{N_{tot}}{LD}\pi B_{z}\sin\left(\omega t\right)\sum_{j=1}^{K}\sum_{i=1}^{N}{z_{i}\rho }_{j}^{2}\Delta z_{i}\Delta \rho_{j}$$

Due to the small thickness of the copper wire, $d=\Delta z_{i}=\Delta \rho_{j}$, for each turn of the PUCs 1, 2, 3 and 4, we can approximate each sum of the expression (A4) with the corresponding integral:

(A5) $$\Phi_{ZDC}^{i}\left(t\right)=\frac{N_{tot}}{LD}\pi B_{z}\sin\left(\omega t\right)\int_{R_{1}}^{R_{2}}\int_{z_{ci}-\frac{L}{2}}^{z_{ci}+\frac{L}{2}}z\rho^{2}dzd\rho =\frac{{\pi N}_{tot}B_{z}}{3D}z_{ci}\left(R_{2}^{3}-R_{1}^{3}\right)\sin\left(\omega t\right)$$

where $z_{ci}$ is the center on the z-axis of each PUC. Given the fact that PUCs 1, 2, 3 and 4 are identical (same total turns, N_tot_, length, L, inner, $R_{1}$ and outer, $R_{2}$, radius) and that $z_{c4}=-z_{c1}$ and $z_{c3}=-z_{c2}$, from expression (A5), we have that $\Phi_{ZDC}^{1}\left(t\right)=-\Phi_{ZDC}^{4}\left(t\right)$ and $\Phi_{ZDC}^{2}\left(t\right)=-\Phi_{ZDC}^{3}\left(t\right)$. Thus, from expression (A1), it is obvious that the magnetic flux recorded by the SDC in the presence of an externally applied uniform and linearly varying harmonic magnetic field, $B_{ext}\left(z,t\right)$, is zero, $\Phi_{SDC}\left(t\right)=0$, which means that the inductive voltage is also zero, $V_{AC}\left(t\right)=-d\Phi_{SDC}\left(t\right)/dt=0$. In this way, the only contribution to $V_{AC}(t)$, originates from the magnetization of the sample.

## Appendix B

Here, we prove that the FDC of the two coaxial PUCs rejects the signal that originates from a uniform magnetic field. The magnetic flux recorded by the FDC in the presence of an externally applied uniform harmonic magnetic field, $B_{ext}\left(t\right)=\left(B_{x}\hat{x}+B_{y}\hat{y}+B_{z}\hat{z}\right)\sin\left(\omega t\right)$, where $B_{x}$, $B_{y}$ and $B_{z}$ are constants, is given by the expression

(A6) $$\Phi_{FDC}\left(t\right)=\Phi_{ZDC}^{1}\left(t\right)-\Phi_{ZDC}^{2}\left(t\right)$$

where $\Phi_{ZDC}^{i}\left(t\right)$, with i=1,2 being the magnetic fluxes being recorded by the respective PUC due to the $B_{ext}\left(t\right)$ and the negative sign due to the opposite winding. The geometric characteristics are shown in Figure 1b for the FDC and in Figure 1a and Figure 2b,c for the ZDC. In order to calculate the magnetic fluxes, $\Phi_{ZDC}^{i}\left(t\right)$, we must first calculate the magnetic flux recorded by each individual 1-turn of the PUCs 1 and 2, $\Phi_{1-turn}^{i}\left(\rho_{j},z_{i},t\right)$, where $z_{i}$ and $\rho_{j}$ are its position and radius, respectively. Only the z component contributes to the magnetic flux, $\Phi_{1-turn}^{i}\left(\rho_{j},z_{i},t\right)$, thus

(A7) $$\Phi_{1-turn}^{i}\left(\rho_{j},z_{i},t\right)=∯B_{ext}\left(t\right)\cdot da=\int_{0}^{2\pi }\int_{0}^{\rho_{j}}B_{z}\sin\left(\omega t\right)\rho d\rho d\varphi =\pi B_{z}\sin\left(\omega t\right)\rho_{j}^{2}$$

The total magnetic flux recorded by the PUCs 1 and 2 due to the $B_{ext}\left(t\right)$ will be the sum of the magnetic fluxes, $\Phi_{1-PUC}^{i}\left(\rho_{j},z_{i},t\right)$, of each turn:

(A8) $$\Phi_{ZDC}^{i}\left(t\right)=\sum_{j=1}^{K}\sum_{i=1}^{N}\Phi_{1-PUC}^{i}\left(\rho_{j},z_{i},t\right)=\pi B_{z}\sin\left(\omega t\right)\sum_{j=1}^{K}\sum_{i=1}^{N}\rho_{j}^{2}$$

From the combination of the expressions (4), (9) and (10), which consist of the mathematical modeling of the ZDC, it follows that $\frac{N_{tot}}{LD}\Delta z_{i}\Delta \rho_{j}=1$; thus, the expression (A8) becomes

(A9) $$\Phi_{ZDC}^{i}\left(t\right)=\frac{N_{tot}}{LD}\pi B_{z}\sin\left(\omega t\right)\sum_{j=1}^{K}\sum_{i=1}^{N}\rho_{j}^{2}\Delta z_{i}\Delta \rho_{j}$$

Due to the small thickness of the copper wire, $d=\Delta z_{i}=\Delta \rho_{j}$, for each turn of PUCs 1 and 2, we can approximate each sum of the expression (A9) with the corresponding integral:

(A10) $$\Phi_{ZDC}^{i}\left(t\right)=\frac{N_{tot}}{LD}\pi B_{z}\sin\left(\omega t\right)\int_{R_{1}}^{R_{2}}\int_{z_{ci}-\frac{L}{2}}^{z_{ci}+\frac{L}{2}}\rho^{2}dzd\rho =\frac{{\pi N}_{tot}B_{z}}{3D}\left(R_{2}^{3}-R_{1}^{3}\right)\sin\left(\omega t\right)$$

Given the fact that PUCs 1 and 2 are identical (same total turns, N_tot_, length, L, inner, $R_{1}$ and outer, $R_{2}$, radius), from expression (A10) we have that $\Phi_{ZDC}^{1}\left(t\right)=\Phi_{ZDC}^{2}\left(t\right)$. Thus, from the expression (A6) it is obvious that the magnetic flux recorded by the FDC in the presence of an externally applied uniform magnetic field, $B_{ext}\left(t\right)$, is zero, $\Phi_{FDC}\left(t\right)=0$, which means that the inductive voltage is also zero, $V_{AC}\left(t\right)=-d\Phi_{FDC}\left(t\right)/dt=0$. In this way, the only contribution to $V_{AC}(t)$ originates from the magnetization of the sample.
